# A tumor microenvironment-related risk model for predicting the prognosis and tumor immunity of breast cancer patients

**DOI:** 10.3389/fimmu.2022.927565

**Published:** 2022-08-18

**Authors:** Shengkai Geng, Yipeng Fu, Shaomei Fu, Kejin Wu

**Affiliations:** The Obstetrics and Gynecology Hospital of Fudan University, Shanghai, China

**Keywords:** breast cancer, tumor microenvironment, prognostic factor, tumor immunity, therapeutic efficiency

## Abstract

**Background:**

This study aimed to construct a tumor microenvironment (TME)-related risk model to predict the overall survival (OS) of patients with breast cancer.

**Methods:**

Gene expression data from The Cancer Genome Atlas was used as the training set. Differentially expressed gene analysis, prognosis analysis, weighted gene co-expression network analysis, Least Absolute Shrinkage and Selection Operator regression analysis, and Wald stepwise Cox regression were performed to screen for the TME-related risk model. Three Gene Expression Omnibus databases were used to validate the predictive efficiency of the prognostic model. The TME-risk-related biological function was investigated using the gene set enrichment analysis (GSEA) method. Tumor immune and mutation signatures were analyzed between low- and high-TME-risk groups. The patients’ response to chemotherapy and immunotherapy were evaluated by the tumor immune dysfunction and exclusion (TIDE) score and immunophenscore (IPS).

**Results:**

Five TME-related genes were screened for constructing a prognostic signature. Higher TME risk scores were significantly associated with worse clinical outcomes in the training set and the validation set. Correlation and stratification analyses also confirmed the predictive efficiency of the TME risk model in different subtypes and stages of breast cancer. Furthermore, immune checkpoint expression and immune cell infiltration were found to be upregulated in the low-TME-risk group. Biological processes related to immune response functions were proved to be enriched in the low-TME-risk group through GSEA analysis. Tumor mutation analysis and TIDE and IPS analyses showed that the high-TME-risk group had more tumor mutation burden and responded better to immunotherapy.

**Conclusion:**

The novel and robust TME-related risk model had a strong implication for breast cancer patients in OS, immune response, and therapeutic efficiency.

## Introduction

In recent years, breast cancer has become the most frequently diagnosed cancer for women globally (1–3). In China, breast cancer, with the highest incidence, is the leading cause of cancer-related mortality in females (4, 5). With increasing importance attached to individualized precision therapy, the traditional tumor–node–metastasis (TNM) stage system and molecular typing PAM50 had been challenged since the prognosis or treatment response of patients at the same stage or with the same molecular subtype could vary substantially due to the heterogeneity of the tumor (6). Thus, the average treatment benefits for unselected patients are low, motivating tumor biology-based selection strategies *via* gene expression assays (GEAs) including Oncotype DX (7), MammaPrint in luminal early breast cancer (8–11), and Fudan University Shanghai Cancer Center gene panel in metastatic triple-negative breast cancer (TNBC) (12). However, the strategies mentioned above were strictly refined to certain molecular subtypes; this called for a universal method to stratify breast cancer patients across different molecular subtypes.

The tumor microenvironment (TME) plays an essential role in the occurrence and development of cancer (13–15), which is reflected by the various immune cells, stromal cells, cytokines, and extracellular matrix molecules existing in the microenvironment. Accumulating evidence had demonstrated that immune cells, as components of TME, were significantly associated with a breast cancer patient’s therapy efficiency and prognosis (16, 17). In addition, research (18–20) revealed that stromal cells recruited by cancer cells from nearby endogenous host stroma were significantly associated with events such as tumor angiogenesis, proliferation, invasion, and metastasis. Furthermore, the extensive cross-talk between immune and stromal cells had a profound influence on a breast cancer patient’s prognosis (21). To date, TME was increasingly considered as a target for combination therapy in patients with breast cancer (21). Previous studies (22–27) had focused on the involvement of TME in the combination with conventional therapies to boost therapeutic responses and prolong the survival of breast cancer patients. However, few studies reported that TME could also be used as a prognostic factor for breast cancer patients, let alone the significance of TME in predicting tumor immunity and therapeutic efficiency for breast cancer patients. Therefore, with proper evaluation, it is reasonable to dig further into the predictive factor of TME in breast cancer patients.

In 2013, Yoshihara *et al.* constructed a new algorithm called ESTIMATE algorithm (28) to infer the proportion of stromal and immune cells in tumor samples. Previous studies had proved the ESTIMATE algorithm to be an effective tool in predicting the TME status (29). In this study, we used ESTIMATE algorithm (28) to calculate the immune, stromal, and ESTIMATE scores and to evaluate the TME status in breast cancer. Gene expression data from The Cancer Genome Atlas (TCGA) were used to establish a TME risk prognostic model based on TME-related genes. Gene Expression Omnibus (GEO) databases were used to validate the predictive efficiency of the prognostic model. Gene set enrichment analysis (GSEA) method was used to explore the possible immune function involved in the TME risk model. Furthermore, the correlation between the TME risk model and tumor mutation burden as well as immunotherapy efficiency was also investigated.

## Materials and methods

### Data collection

The RNA-normalized sequencing data (1,053 breast cancer tissues and 111 matched normal tissues, fragment per kilobase per million) and corresponding clinical information (including clinical characteristics and tumor mutation status) of patients with breast cancer were downloaded from the TCGA database (19 patients without corresponding clinical information were excluded). Normalized gene expression data of GSE158309, GSE17705, and GSE31448 were downloaded from the GEO database. Genome-wide co-expression analysis was performed to investigate potential TME- and prognosis-related genes based on the TCGA database. The datasets from GEO database were independently used for external validation. Immune score, stromal score, and tumor purity were calculated using the ESTIMATE algorithm (28) provided in the R package “estimate”.

### Development of the TME-related prognostic gene signature

Differentially expressed gene (DEG) analysis was performed using the edgeR filtering method included in the “Limma” R package. TME-related DEGs were defined as genes whose false discovery rate (FDR) value was <0.05 and log_2_ (fold change) was >1. Weighted gene co-expression network analysis (WGCNA) was performed to recognize gene modules related to the immune and stromal scores. Gene modules with a correlation coefficient >0.5 were considered as strong TME-correlated modules. Univariate Cox regression analysis was used to recognize prognostic genes (*p* < 0.05, two-tailed) for patients with breast cancer in the TCGA dataset. The intersections of the immune- and stromal-related genes screened by DEGs, WGCNA, and univariate Cox regression analyses were all inputted in the Least Absolute Shrinkage and Selection Operator (LASSO) regression analysis to identify the hub genes. The combination of two hub genes was inputted into the Wald stepwise Cox regression to develop the TME risk model. In this process, the model with minimal Akaike information criterion (AIC) value was determined as the final mode. The signature was defined as TME risk score = ∑coefficient-mRNA_i_ × expression of mRNA_i_. Receiver operating characteristic curve (ROC) analysis was used to determine the optimal cutoff value for the high- and low-TME-risk groups in SPSS version 25 (SPSS Inc.).

### Survival and immune analysis for low- and high-TME-risk groups

ROC analysis was used to determine the optimal cutoff value of the TME risk score for patients’ overall survival (OS). After the patients from the TCGA dataset were divided by the cutoff value of the TME risk score, we used t-distributed stochastic neighbor embedding (t-SNE) and principal component analysis (PCA) to evaluate the discrimination of the model. The association of clinicopathologic characteristics and stromal–immune scores between low- and high-TME-risk groups was analyzed using a two-sided chi-square test. The survival curves were determined by the Kaplan–Meier analysis and compared by the log-rank test. The Cox proportional hazards regression model was used to perform univariate and multivariate analyses, and *P <*0.05 (two-tailed) was considered statistically significant. We used the time-dependent area under the receiver operating characteristic curve (AUC) and C statistics to evaluate the predictive power of TME risk for OS. Calibration plots were used to evaluate the discriminative ability and accuracy of the models. Stage- and subtype-stratified analyses of prognostic significance of TME risk in patients with breast cancer were performed. The HUMAN PROTEIN ATLAS (HPA) database and Gene Expression Profiling Interactive Analysis (GEPIA) were used to evaluate the immunohistochemical (IHC) staining and the prognostic significance of the TME risk signature. The expression of the human leukocyte antigen (HLA) gene family and immune checkpoints (30, 31) was evaluated in both low- and high-TME-risk groups. The population abundance of tissue-infiltrating immune and stromal immune and stromal cells were calculated with different methods, including TIMER (32), CIBERSORT (33), and Xcell (34) algorithms (available at TIMER2.0 website, http://timer.comp-genomics.org/). The correlation between the hub genes and different immune cell infiltration was evaluated using the Wilcoxon test.

### Function enrichment and tumor mutation status analysis in the low- and high-TME-risk groups

The biological function related to TME risk was investigated using the GSEA method. FDR *q*-value <0.05 and |normalized enriched score (NES)| >1 were considered significantly enriched. Then, we evaluated the function enrichment of the identified TME risk genes using Gene Ontology (GO) function analysis (including biological process, cellular component, and molecular function) and Kyoto Encyclopedia of Genes and Genomes (KEGG) analysis. After downloading the breast cancer patient’s tumor mutation information from the TCGA database, different mutation types were evaluated in both the high- and low-TME-risk groups. In addition, tumor mutational burden (TMB) and mutation counts were calculated to find the potential correlation between TME risk and tumor mutation status. Stratified analysis was performed to investigate the tumor mutation difference in different subtypes. The Spearman method was used to calculate the correlation coefficient, and *P <*0.05 (two-tailed) was considered statistically significant.

### Prediction of immune escape and immunotherapy efficiency in patients in groups with different TME risks

We used the pRRophetic algorithm in R language to evaluate the 50% inhibiting concentration (IC50) value of the 88 drugs in the low- and high-TME-risk groups. The potential response of patients to immunotherapy was inferred by the immunophenscore (IPS) (35) (downloaded from The Cancer Immune Group Atlas, TCIA) and tumor immune dysfunction and exclusion (TIDE) score.

## Results

### Correlation of the clinical features of patients with breast cancer with immune/stromal scores

A total of 1,164 samples (111 normal tissue samples and 1,053 breast cancer samples) in the TCGA database were included in our study to calculate the immune and stromal scores. The stromal scores of these patients ranged from -2,033.4 to 2,083.4, and the immune scores ranged from -1,162.0 to 3,638.8. There were no significant differences between normal and breast cancer tissues in relation to immune scores, while normal tissue had higher stromal scores than breast cancer samples. The correlation of the clinicopathological characteristics in breast cancer patients between different groups is presented in **Supplementary Figure S1**. In our study, the immune and stromal scores had no significantly different distribution in the old/young patients, T stage, N stage, and TNM stage. In addition, the survival analysis showed that patients with higher immune scores had a more favorable OS than those with lower immune scores.

### Development of a prognostic TME risk signature with the TCGA cohort

**Figure 1:**
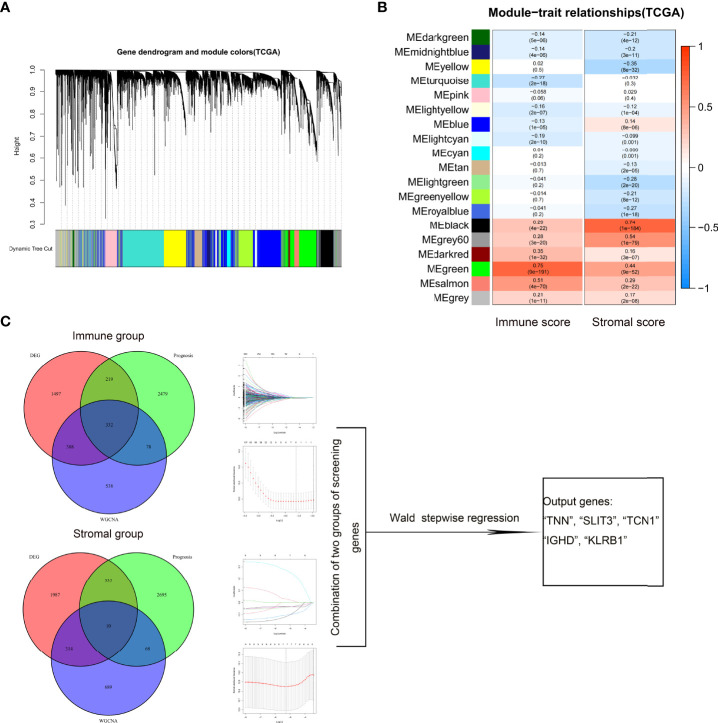
Screening for TME-related genes. Gene modules identified by WGCNA **(A)**. Correlation between gene modules and immune/stromal scores **(B)**. Venn plot showing the number of intersection genes among DEG analysis, prognosis, and WGCNA analysis in immune/stromal scores **(C)**. These genes were further inputted into LASSO analysis, respectively. The genes selected at minimum error values of lambda were used for analysis. Combinations of two groups of screening genes were inputted further into Wald stepwise regression. The final calculation result is displayed in the output table. TME, tumor microenvironment; WGCNA, weighted gene co-expression network analysis; DEG, differentially expressed gene; LASSO, Least Absolute Shrinkage and Selection Operator.

Divided by the median value of immune/stromal scores, the DEG analysis between the low- and high-immune/stromal score groups was performed using the edgeR filtering method. In our study, six was selected as the optimal soft threshold of WGCNA. As shown in **Figure 1**, 19 co-expressed gene modules were recognized in relation to immune/stromal scores. The green and salmon had a strong correlation with the immune score, whereas the gray and black were associated with the stromal score. Univariate Cox regression analysis was used to recognize the prognostic genes for the groups with different immune/stromal scores. The Venn plot showed the intersection of DEGs, TME-risk-correlated gene modules, and prognostic genes. After the LASSO–Cox regression analysis, IGHA1, PIGR, APOBEC3D, IGHD, KLRB1, and MATK were selected from the immune score group, whereas MEOX1, COL12A1, HSD11B1-AS1, TNN, SLIT3, TCN1, and CPXM1 were selected from stromal score group. A combination of two groups of screening genes was further inputted into Wald stepwise regression analysis to develop the final model (the result is presented in **Supplementary Table S1**). With the minimal AIC (AIC = 1,546.21), the final model was as follows: TME risk = SLIT3 * 0.329 - TNN * 0.11- TCN1 * 0.051 – IGHD * 0.075 - KLRB1 * 0.164.

### Correlation and stratification analyses of TME risk and validation of the prognostic model

**Figure 2:**
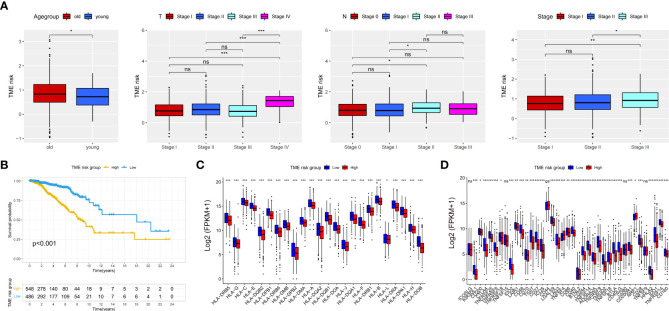
Difference analysis of TME risk scores in age (age over 40 years old was defined as the old group, and age up to 40 years was defined as the young group), T stage, N stage, and TNM stage **(A)**. Kaplan–Meier analysis between low- and high-TME-risk groups **(B)** (the low- and high-TME-risk groups were divided by the optimal cutoff value). Difference analysis for the expression of HLA family **(C)** and immunecheckpoint genes **(D)** between low- and high-TME-risk groups. TME, tumor microenvironment. The statistical difference was compared by pairwise comparisons using Wilcoxon test. Significance: *P < 0.05; **P < 0.01; ***P < 0.001. ns, not significant.

**Figure 3:**
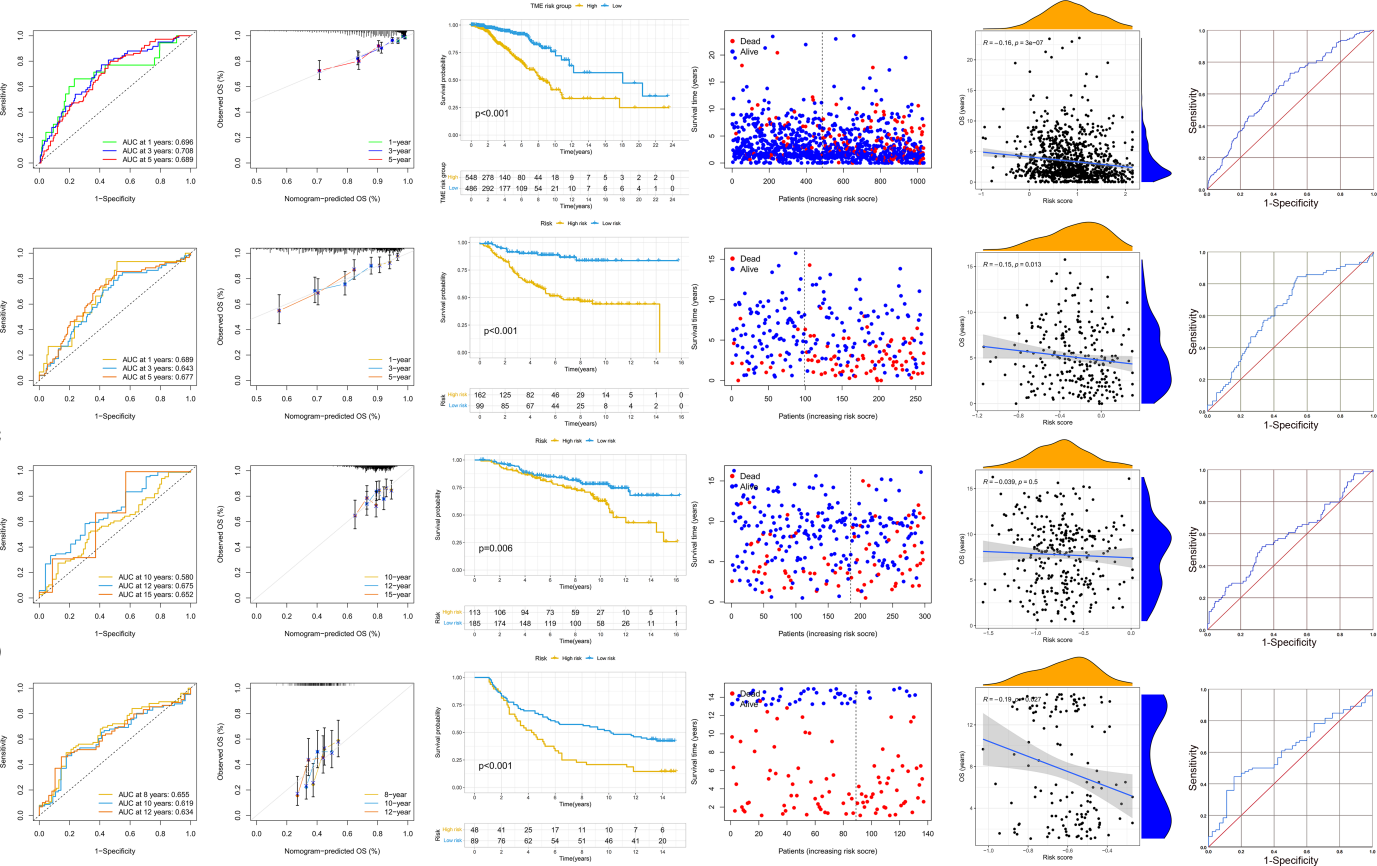
AUC analysis, calibration analysis, Kaplan–Meier analysis, correlation between TME risk scores, and patients’ OS and ROC analysis in the training set **(A)**, validation set [GSE31448, all subtypes of breast cancer patients **(B)**], HR-positive breast cancer patients [GSE17705 **(C)**], and early-stage breast cancer patients [GSE158309 **(D)**]. AUC, area under the curve; TME, tumor microenvironment; OS, overall survival; ROC, receiver operating characteristic curve; HR, hormone receptor; Her-2, human epidermal growth factor receptor 2.

In our study, patients from TCGA were divided into low- and high-TME-risk groups by the optimal cutoff value of TME risk score (1.167) for breast cancer patient’s OS. The PCA and t-SNE analyses demonstrated that patients with different TME risk scores were well separated in two directions (shown in **Supplementary Figures S2A, B**). The correlation between the patients’ clinicopathological characteristics and TME risk scores is shown in **Figure 2**. In our study, we found that higher TME risk scores were associated with higher age, higher T stage, and higher TNM stage. The Kaplan–Meier (KM) analysis showed that the low-TME-risk group of patients with breast cancer had a more favorable clinical outcome (*P* < 0.001). The AUC (for predicting the patients’ OS of 1, 3, and 5 years, this was 0.696, 0.708, and 0.689, respectively) and the calibration plot analyses both confirmed the predictive ability of TME risk score (shown in **Figure 3A**). The survival analysis showed a significantly negative correlation between the TME risk score and patients’ OS. The ROC analysis showed that the C statistics of TME risk was 0.64 (95%CI: 0.592–0.688, *P* = 0.024). Furthermore, the stage-stratified analysis showed that the TME risk score was identified as a prognostic factor for all breast cancer stages (*P* =0.037 for stage I patients, *P <*0.001 for stage II patients, and *P <*0.001 for stage III patients; shown in **Supplementary Figures S3A–C**). In addition, the subtype-stratified analysis showed that HR-positive and TNBC patients with lower TME risk scores had a more favorable OS (*P <*0.001 and *P* =0.008, respectively; shown in **Supplementary Figures S3D–F**). The validation was performed on three GEO datasets (GSE 31448, GSE158309, and GSE17705). The KM analysis in all datasets confirmed the significant association between TME risk and patients’ OS. As shown in **Figure 3B**, the GSE31448 dataset proved the TME risk score prognostic ability in all subtypes of breast cancer patients. The AUC for predicting the patients’ OS of 1, 3, and 5 years was 0.689, 0.643, and 0.677, respectively. To evaluate the long-term outcome prediction of TME risk score, GSE17705 (ER-positive subtype breast cancer patients) and GSE158309 (early breast cancer patients) datasets were enrolled in our study. Both datasets confirmed the association between the TME risk score and the patients’ OS. The AUC for predicting the patients’ OS of 10, 12, and 15 years was 0.580, 0.675, and 0.652 in ER-positive breast cancer patients and that of 8, 10, and 12 years was 0.655, 0.619, and 0.634 in early breast cancer patients, respectively. Similarly, good concordance was observed between the model predicted and the actual observations in three calibration curves. The C statistics of the three datasets are presented in **Figure 3**, showing that TME risk score had a good predictive effect (GSE31448—0.639, 95%CI: 0.565–0.713, *P* = 0.038; GSE17705—0.597, 95%CI: 0.51–0.663, *P* = 0.039; GSE158309—0.609, 95%CI: 0.513–0.706, *P* = 0.049). Furthermore, we used HPA database and GEPIA to evaluate the IHC staining and the prognostic significance of the TME risk signature. The KM analysis, along with the typical IHC staining of SLIT3, TNN, IGHD, and KRRB1, showed that the expression was significantly different between normal and breast cancer tissues and confirmed the prognostic effect of the TME risk signature. The UMAP results of single-cell sequencing (shown in **Supplementary Figure S5**) showed that SLIT3 and TNN were mainly expressed in stromal cells, while IGHD and KLRB1 were mainly enriched in immune cells. In addition, TCN1 was found to be expressed mainly in breast glandular cells. Taken together, our TME risk model integrated the stromal–immune signature, further confirming that breast cancer prognosis was significantly associated with the tumor microenvironment.

### Analysis of immune and functional enrichment studies between the low- and high-TME-risk groups

A total of 24 HLA-related genes and 39 immune checkpoints between the low- and high-TME-risk groups were investigated in our study. As shown in **Figures 2C, D**, all HLA family genes and 34 immune checkpoints were significantly different between the low- and high-TME-risk groups as evaluated by the Wilcoxon test. Compared with the high-TME-risk group, most of the immune-related genes in the low-TME-risk group were upregulated, except the TNFSF4 and NRP1 immune checkpoints. To further explore the possible mechanisms underlying the differential expression of immune-associated genes between the low- and high-TME-risk groups, we performed the GSEA analysis with annotations based on the GO and KEGG gene databases. In total, 50 of the most significant enrichment results (|NES| >1 and FDR value <0.05) are presented in **Supplementary Figure S4**. We found that many biological processes related to immune response functions, including proliferation, migration, and infiltration of immune cells, inflammatory responses, chemokine activities, cellular defense responses, and leukocyte migration, were significantly associated with the low-TME-risk group. In addition, a clear inverse correlation was found between the TME risk score and the immune/stromal score (shown in **Supplementary Figures S5A, B**), while the tumor purity score was in accordance with the TME risk tendency (shown in **Supplementary Figure S5C**). The hub genes’ expression had a significantly distinct distribution between the low- and high-TME-risk groups (shown in **Supplementary Figures S5D–H**).

**Figure 4:**
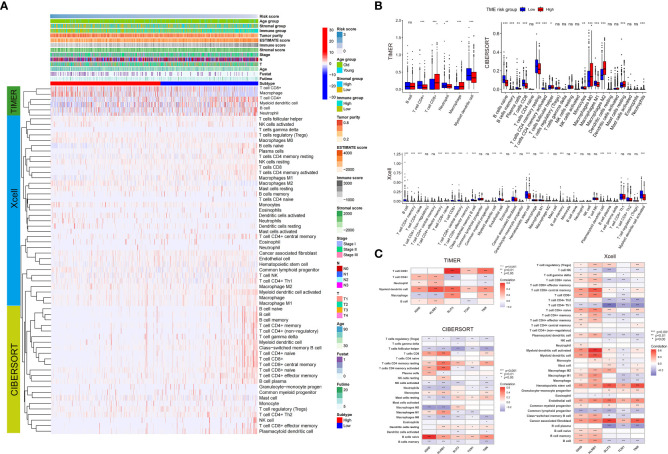
Landscape of immune and stromal cell infiltrations between the low- and high-TME-risk groups **(A)**. Difference analysis of immune and stromal cell infiltrations (analyzed by TIMER, Xcell, and CIBERSORT methods) between the low- and high-TME-risk groups **(B)**. Correlation analysis between five screening prognostic genes and immune/stromal cell infiltration analyzed by TIMER, Xcell, and CIBERSORT methods **(C)**. The statistical difference was compared using Wilcoxon test. Significance: *P < 0.05; **P < 0.01; ***P < 0.001; ns, not significant. TME, tumor microenvironment.

We then investigated the distribution of infiltrating immune cells as inferred by TIMER, CIBERSORT, and xCell between the low- and high-TME-risk groups (shown in **Figure 4**). Our study revealed that most of the immune and stromal cells, including B cells, T cells CD4+, T cells CD8+, and myeloid dendritic cells, increased in the low-TME-risk group. However, macrophages (especially macrophage II) infiltrated more in the high-TME-risk group, while there was no uniform result in terms of neutrophils. Meanwhile, we did not find significant differences in T cells CD4+ Th1/Th2 and NK cells between the two groups.

### The correlation between TME risk and tumor mutation status

**Figure 5:**
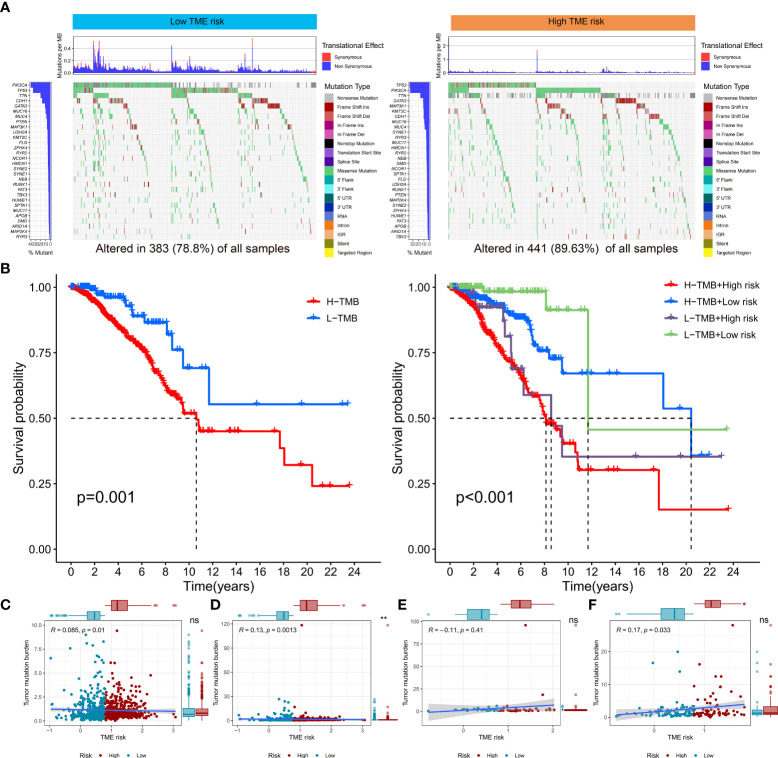
Landscape of TMB and tumor mutation status in low- and high-TME-risk groups **(A)**. Kaplan–Meier analysis between low- and high-TME-risk groups combined with low- and high-TMB groups **(B)**. Association between breast cancer patient’s TMB [**(C)** all patients, **(D)** HR-positive breast cancer patients, **(E)** Her-2 positive breast cancer patients, and **(F)** TNBC patients] and distribution in groups with different TME risks. TMB, tumor mutation burden; TME, tumor microenvironment; HR, hormone receptor; Her-2, human epidermal growth factor receptor 2; TNBC, triplenegative breast cancer. The statistical difference was compared by pairwise comparisons using Wilcoxon test. Significance: *P < 0.05; **P < 0.01; ***P < 0.001. ns, not significant.

We downloaded the breast cancer patient’s tumor mutation information from the TCGA database to evaluate its correlation with the TME risk score. As shown in **Figure 5**, more somatic mutations were presented in the high-TME-risk group, while the maftools analysis results showed no significant differences between the low- and high-TME-risk groups in terms of the mutation frequencies of specific genes. Furthermore, the KM analysis revealed that the combination of TMB and TME risk had profound effects on patients’ prognosis, and patients with high TME risk and high TMB tended to have the worst clinical outcome. What is more, the subtype-stratified analysis showed a clear positive association between TME risk score and TMB in ER-positive and TNBC breast cancer patients, which might partly reflect the connection with patients’ prognosis.

### Immune escape and immunotherapy efficiency between low- and high-TME-risk groups

**Figure 6:**
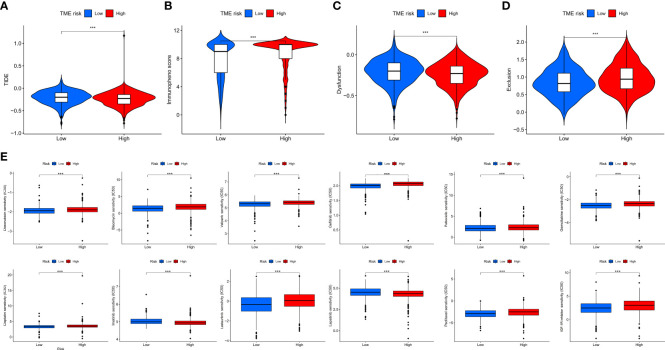
TIDE analysis **(A)**, IPS analysis **(B)**, immune dysfunction analysis **(C)**, and immune exclusion analysis **(D)** between low- and high-TME-risk group. Difference analysiss **(E)** of commonly used clinical drugs’ chemotherapeutic responses including doxorubicin, bleomycin, veliparib, gefitinib, palbociclib, gemcitabine, cisplatin, imatinib, lestaurtinib, lapatinib, paclitaxel, and IGF-1R inhibitor between low- and high-TME-risk groups. Statistical difference was compared using Wilcoxon test. Significance: *P < 0.05; **P < 0.01; ***P < 0.001. ns, not significant; TIDE, tumor immune dysfunction and exclusion; IPS, immunophenscore; TME, tumor microenvironment.

In our study, we used the IC50 value of the 88 drugs to evaluate the TME risk score as a predictive factor for the response of breast cancer patients to therapies (including chemotherapy, targeted therapy, and immunotherapy). Several commonly used clinical drugs are presented in **Figure 6**. We found that patients with a higher TME risk score had lower TIDE scores and higher IPS scores. These results suggested that patients in the high-TME-risk group were more likely to respond better to immunotherapy. In accordance with the TIDE score, immune dysfunction and exclusion analysis also showed that patients with higher TME risk scores might benefit more from immunotherapy. In addition, the IC50 analysis showed that patients in the high-TME-risk group might be more sensitive to imatinib and lapatinib, while patients in the low-TME-risk group might be more sensitive to palbociclib, paclitaxel, cisplatin, veliparib, *etc.*

## Discussion

In our study, we constructed a novel, easy-to-use, and effective TME risk score system based on immune and stromal scores for the prediction of breast cancer patient’s OS. First, our results showed that the TME risk was an independent prognostic factor for all breast cancer patients. With the stratified analysis and external validation of GEO datasets, we confirmed the predictive efficiency of TME risk in all stages of breast cancer patients, especially in early stage, TNBC, and HR-positive breast cancer patients. In addition, our study showed that patients in the low-TME-risk group presented higher levels of immune and stromal cell infiltration, higher immunogenicity, lower tumor purity, and lower somatic mutation status than patients in the high-TME-risk group. Furthermore, a positive association was found between TME risk and TMB status, the combination of which might have a significant prognostic value for breast cancer patients. Finally, the TIDE and IPS score analyses demonstrated that TME risk could be a potential biomarker for predicting immunotherapeutic response for breast cancer patients.

The TME risk score was constructed based on the immune and stromal scores calculated by the ESTIMATE algorithm. Previous studies had demonstrated the prognostic value of this algorithm in many diseases, such as gastric adenocarcinoma (36, 37), colon cancer (38), lung adenocarcinoma (39), clear cell renal cell carcinoma (40), *etc.* The effect of tumor-infiltrating lymphocytes on breast cancer patients’ clinical outcomes was well established in previous research (41, 42). In accordance with the previous studies, we found that patients with better prognosis had higher immune scores, while no significant overall association of clinical characteristics of breast cancer with stromal score alone was observed. However, the TME risk score, calculated based on the combination of immune and stromal scores, was identified to be significantly associated with breast cancer patients’ T stage, TNM stage, and OS, indicating that we should not separately look at the impact of immune and stroma on the prognosis of breast cancer. In addition, the stratified analysis revealed that the TME risk was an independent prognostic factor for patients in different TNM stages. For HR-positive patients and TNBC, the high TME risk was significantly associated with worse clinical outcomes. Furthermore, the robust performance of the TME risk model for long-term prognosis in HR-positive and early breast cancer patients was confirmed by the AUC analyses in three independent GEO datasets. The calibration curves and correlation analysis also showed good concordance between the model predicted and the actual observations. Additionally, the analysis of drug sensitivity revealed that the TME risk model influenced the patients’ drug response to chemotherapy and targeted therapy. The IC50 analysis showed that patients in the high-TME-risk group might be more sensitive to imatinib and lapatinib, while patients in the low-TME-risk group might be more sensitive to palbociclib, paclitaxel, cisplatin, veliparib, *etc.* Taken together, our results revealed that the TME risk prognostic model might aid physicians in making clinical therapy decisions and guiding the long-term follow-up to let breast cancer patients gain survival benefits.

Maintaining a good predictive value, our finial prognostic model included only five genes selected based on the stromal and immune scores, which could reduce the patients’ unnecessary waste. TNN, known as Tenascin-N or -W, was involved in neurite outgrowth and cell migration in hippocampal explants. In tumors, it stimulates angiogenesis by the elongation, migration, and sprouting of endothelial cells (43). In terms of breast cancer, it was significantly downregulated in tumor samples and might facilitate tumorigenesis by supporting the migratory behavior of breast cancer cells (44). SLIT3 was involved in the final model, and previous researches had demonstrated that the suppression of SLIT3 might induce tumor proliferation and invasion in many solid tumors such as ovarian cancer (45), hepatocellular carcinoma (46), thyroid cancer (47), and gastric cancer (48). TCN1 (Transcobalamin I, vitamin B12 binding protein, R binder family) encodes a member of the vitamin B12-binding protein family. This family of proteins, alternatively referred to as R binders, is expressed in various tissues and secretions. Previous research had shown that a high expression of TCN1 was a negative prognostic factor in colon cancer and might correlate with the patients’ chemosensitivity (49–51). The TME risk prognostic model also included immune-related genes (KLRB1 and IGHD). Proteins coded by these genes were associated with the immune microenvironment (52) and immune cell infiltration (53) (such as natural killer cells and T cells). In our study, the low-TME-risk group upregulated in many immune checkpoints and increased in most of the immune and stromal cells, including B cells, T cells CD4+, T cells CD8+, and myeloid dendritic cells. In addition, the function analyses (GO and KEGG) revealed that many biological processes related to immune response functions were significantly associated with the low-TME-risk group. Taken together, these might be why patients in the low-TME-risk group had a better prognosis.

Immunotherapy, as an emerging novel treatment modality, is increasingly applied in the treatment of cancer patients (54–56). However, the optimal patient selection who may benefit from immunotherapy remains a great challenge. Recent studies had proved TMB as an emerging biomarker of response to immunotherapy for many cancers (57–59). As shown in **Figure 5**, more somatic mutations were presented in the high-TME-risk group. In addition, the stratified analysis showed a positive correlation between TME risk and TMB, especially in ER-positive and TNBC patients. What is more, the combination of TMB and TME risk had a profound implication for prognosis. Patients with higher TME risk scores and higher TMB tended to have the worse clinical outcome. Our study revealed that the high-TME-risk group might respond better to immunotherapy using TIDE and IPS score analysis, which was in accordance with the tumor mutation status and previous research. In addition, patients in the high-TME-risk group tended to have lower immune cell infiltration and downregulation of HLA and immune checkpoint expressions. It should be noted that the TNFSF4 and NRP1 immune checkpoints were upregulated in the high-TME-risk group. Previous studies had proved that VEGF-A/NRP1 signaling might be associated with breast cancer metastasis (60, 61). The study (62) by Kai Li *et al.* also revealed the oncogenic features of TNFSF4 and specifically demonstrated the potential effects of applying TNFSF4 blockade-based immunotherapies in breast carcinomas. Taken together, our results might suggest potential therapeutic targets and provide novel clinical applications for immunotherapies.

There were several limitations in our studies. Firstly, due to the lack of clinical details of breast cancer patients in public databases (such as menopause status and chemotherapy regimens), we cannot perform a more in-depth stratification analysis between the low- and high-TME-risk groups. Secondly, database information on immune infiltration and stroma status was inferred by TIMER, CIBERSORT, and xCell between the low- and high-TME-risk groups only using the expression data of immune-associated genes.

## Conclusion

In conclusion, our study successfully constructed and validated a novel and robust TME-related prognostic model for breast cancer patients. Furthermore, our predictive model could seek the possibility to find selected patients who would benefit more from anticancer immunotherapy and adjuvant chemotherapy, which would reduce the risk of complications and give the patients better individual treatment guidance.

## Data availability statement

The RNA-seq data and corresponding clinical information were observed from the TCGA (https://portal.gdc.cancer.gov/) and GEO (https://www.ncbi.nlm.nih.gov/geo/). The IPS for breast cancer patients were retrieved from the TCIA (https://tcia.at/home). The accession number(s) can be found in the article/**Supplementary Material**.

## Ethics statement

The studies involving human participants were reviewed and approved by TCGA and GEO databases. The patients/participants provided their written informed consent to participate in this study. Written informed consent was obtained from the individual(s) for the publication of any potentially identifiable images or data included in this article.

## Author contributions

All authors participated in the design, interpretation of the studies, analysis of the data, and review of the manuscript. SG and YF conceived and designed the whole project and wrote the manuscript. SG and YF performed the data analyses. SG, YF, and KW interpreted the data and partook in the discussion. SF and KW revised the final version of the manuscript. All authors contributed to the article and approved the submitted version.

## Funding

This work was supported by the National Natural Science Foundation of China (no. 81902993), Shanghai Health Committee (no. 2020YJZX0204), and Shanghai Shenkang Hospital Development Center (no. SHDC22020209).

## Acknowledgments

We would like to appreciate the TCGA, GEO, HPA, GEPIA, and TCIA database for the availability of the data.

## Conflict of interest

The authors declare that the research was conducted in the absence of any commercial or financial relationships that could be construed as a potential conflict of interest.

## Publisher’s note

All claims expressed in this article are solely those of the authors and do not necessarily represent those of their affiliated organizations, or those of the publisher, the editors and the reviewers. Any product that may be evaluated in this article, or claim that may be made by its manufacturer, is not guaranteed or endorsed by the publisher.

## Supplementary material

Supplementary Figure 1 Difference analysis of immune scores **(A)** and stromal scores **(B)** in normal/tumor, age (age over 40 years old was defined as the old group, age up to 40 years was defined as the young group), T stage, N stage, TNM stage. Statistical difference was compared by pairwise comparisons using Wilcoxon test. Significance: **P* < 0.05; ***P* < 0.01; ****P* < 0.001; ns, not significant. Kaplan-Meier analysis between low and high immune group **(C)**/stromal group **(D)** (low and high immune/stromal group was divided by the average value).

Supplementary Figure 2 The PCA **(A)** and t-SNE **(B)** analysis between low and high TME-risk group. PCA, principal-component analysis; t-SNE, t-distributed stochastic neighbor embedding.

Supplementary Figure 3 Kaplan-Meier analysis between low and high TME-risk group in stage I **(A)**, stage II **(B)**, stage III **(C)**, HR positive breast cancer patients **(D)**, Her-2 positive breast cancer patients **(E)** and TNBC patients **(F)**. TME, tumor microenvironment; HR, hormone receptor; Her-2, human epidermal growth factor receptor 2; TNBC, triple negative breast cancer.

Supplementary Figure 4 Kaplan-Meier analysis and Immunohistochemical staining of the TME-risk signature, including SLIT3 **(A)**, TNN **(B)**, IGHD **(C)**, KLRB1 **(D)**, and TCN1 **(E)**. TME; tumor microenvironment.

Supplementary Figure 5 The UMAP results of single cell sequencing of the TME-risk signature, including SLIT3 **(A)**, TNN **(B)**, IGHD **(C)**, KLRB1 **(D)**, and TCN1 **(E)**. TME, tumor microenvironment.

Supplementary Figure 6 GSEA analysis (including GO and KEGG analysis) between low and high TME-risk groups (top 50 listed biological function in BP **(A)**, CC **(B)**, MF **(C)** and KEGG **(D)**, (FDR q-value <0.05 and |NES| > 1)). GSEA, gene set enrichment analysis; GO, gene ontology; KEGG, Kyoto Encyclopedia of Genes and Genomes.

Supplementary Figure 7 Association between immune scores **(A)**, stromal scores **(B)**, tumor purity **(C)**, and TME-risk scores and their distribution in the low and high TME-risk groups. Association between 5 screening genes (SLIT3 **(D)**, TNN **(E)**, IGHD **(F)**, KLRB1 **(G)**, TCN1 **(H)**) and TME-risk scores and their distribution in the low and high TME-risk groups. TME, tumor microenvironment.
