# Supplementary material for: A tumor microenvironment-related risk model for predicting the prognosis and tumor immunity of breast cancer patients
Source: Front Immunol. 2022 Aug 18;13:927565. doi: 10.3389/fimmu.2022.927565 (PMC9433750; doi:10.3389/fimmu.2022.927565)
Supplement: Supplementary file 1 [file Table_1.docx]

| Supplement table 1. Final wald stepwise regression result | | | | | |
| --- | --- | --- | --- | --- | --- |
| Gene | β | SE | Wald | P | HR |
| TNN | -0.11 | 0.049 | 5.002 | 0.025 | 0.896 |
| SLIT3 | 0.329 | 0.071 | 21.665 | <0.001 | 1.39 |
| TCN1 | -0.051 | 0.026 | 3.856 | 0.049 | 0.95 |
| IGHD | -0.075 | 0.035 | 4.689 | 0.03 | 0.928 |
| KLRB1 | -0.164 | 0.073 | 5.007 | 0.025 | 0.849 |
