# Supplementary figures and images for: A tumor microenvironment-related risk model for predicting the prognosis and tumor immunity of breast cancer patients
Source: Front Immunol. 2022 Aug 18;13:927565. doi: 10.3389/fimmu.2022.927565 (PMC9433750; doi:10.3389/fimmu.2022.927565)

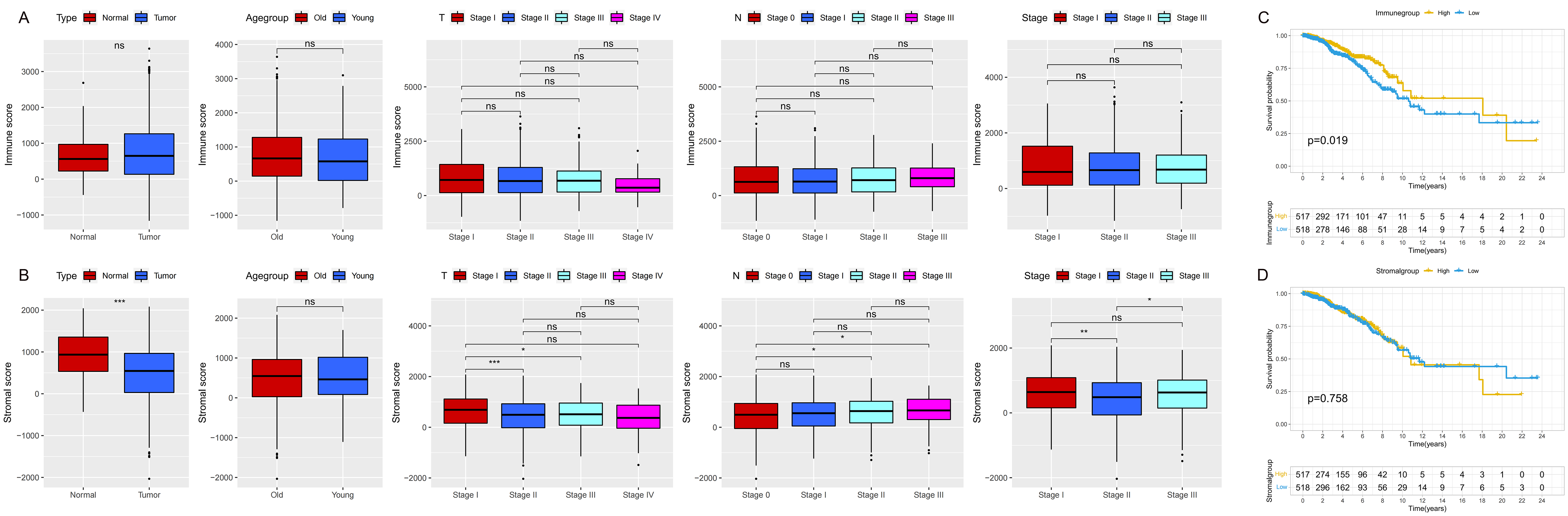

Supplement: Supplementary Figure 1 — Difference analysis of immune scores (A) and stromal scores (B) in normal/tumor, age (age over 40 years old was defined as the old group, age up to 40 years was defined as the young group), T stage, N stage, TNM stage. Statistical difference was compared by pairwise comparisons using Wilcoxon test. Significance: *P < 0.05; **P < 0.01; ***P < 0.001; ns, not significant. Kaplan-Meier analysis between low and high immune group (C)/stromal group (D) (low and high immune/stromal group was divided by the average value). [file Image_1.tif]

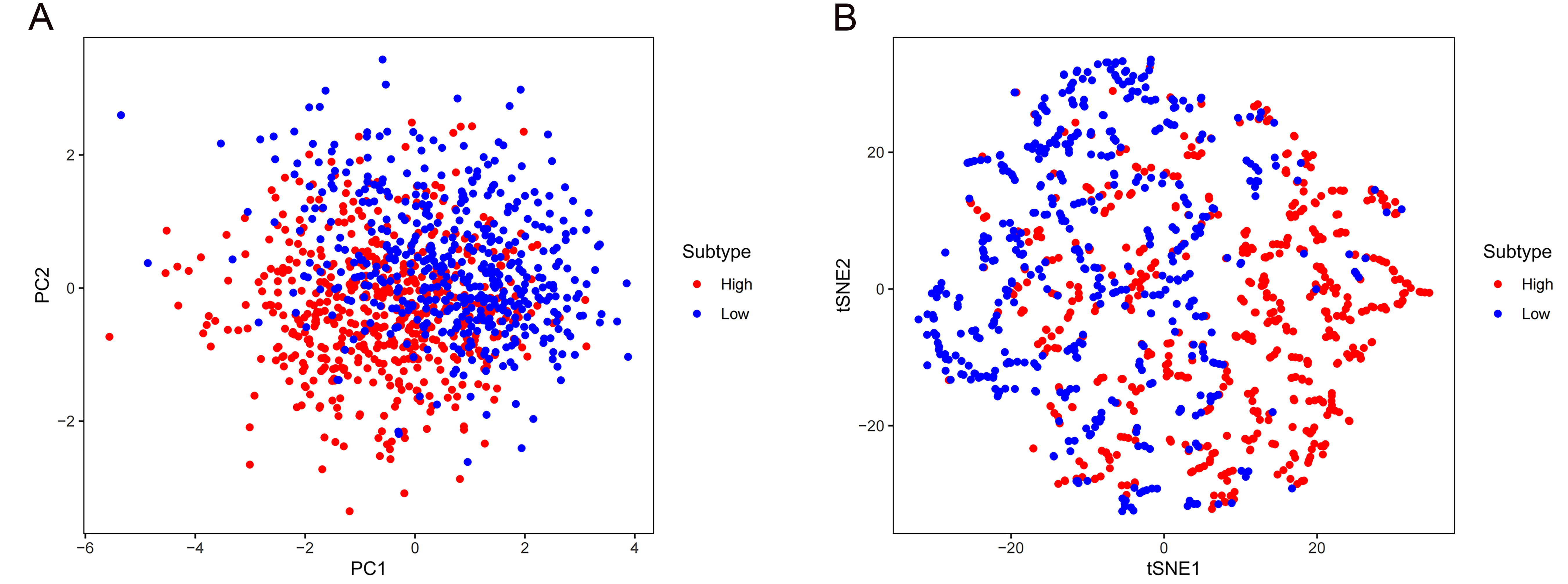

Supplement: Supplementary Figure 2 — The PCA (A) and t-SNE (B) analysis between low and high TME-risk group. PCA, principal-component analysis; t-SNE, t-distributed stochastic neighbor embedding. [file Image_2.tif]

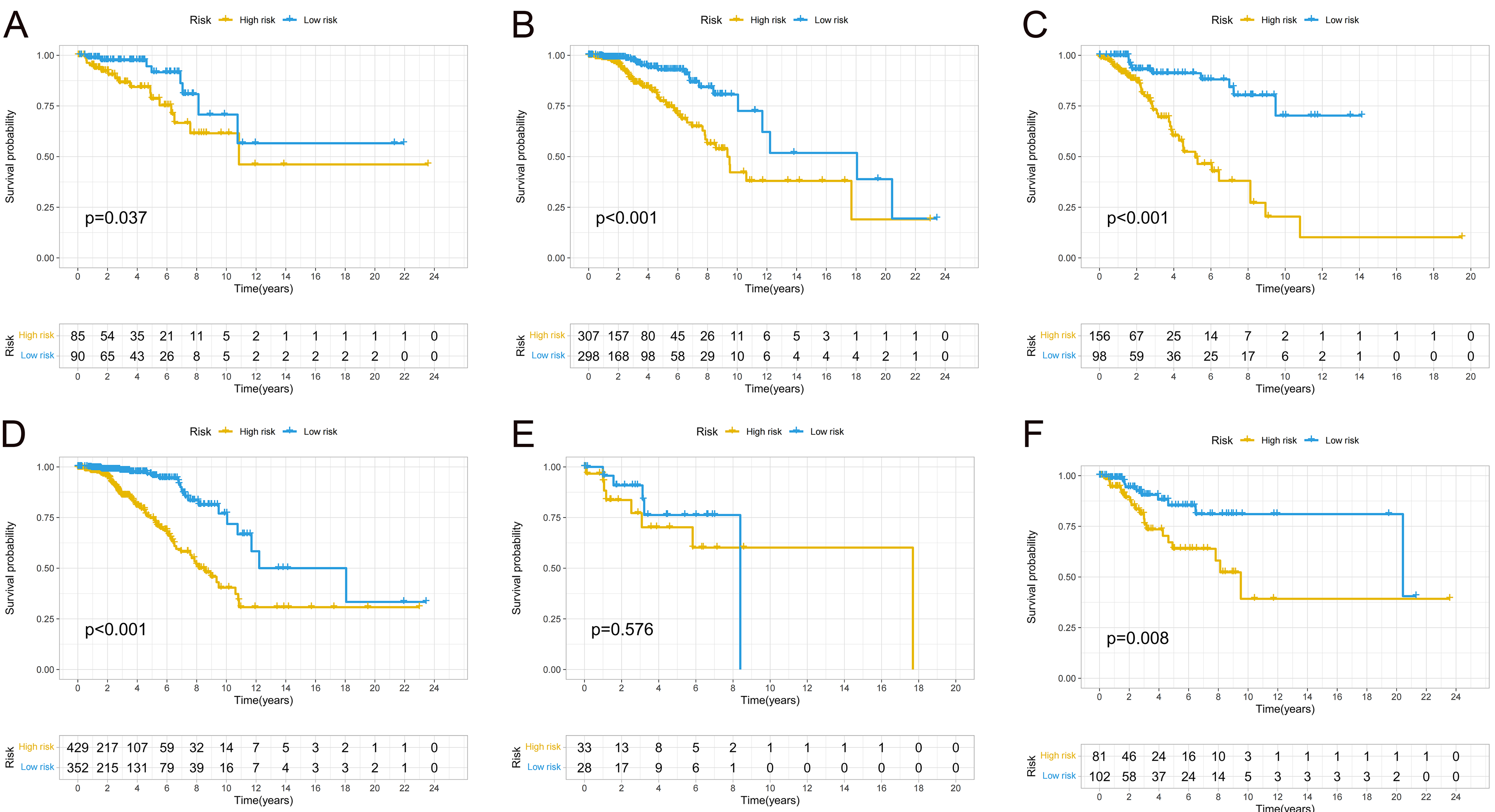

Supplement: Supplementary Figure 3 — Kaplan-Meier analysis between low and high TME-risk group in stage I (A), stage II (B), stage III (C), HR positive breast cancer patients (D), Her-2 positive breast cancer patients (E) and TNBC patients (F). TME, tumor microenvironment; HR, hormone receptor; Her-2, human epidermal growth factor receptor 2; TNBC, triple negative breast cancer. [file Image_3.tif]

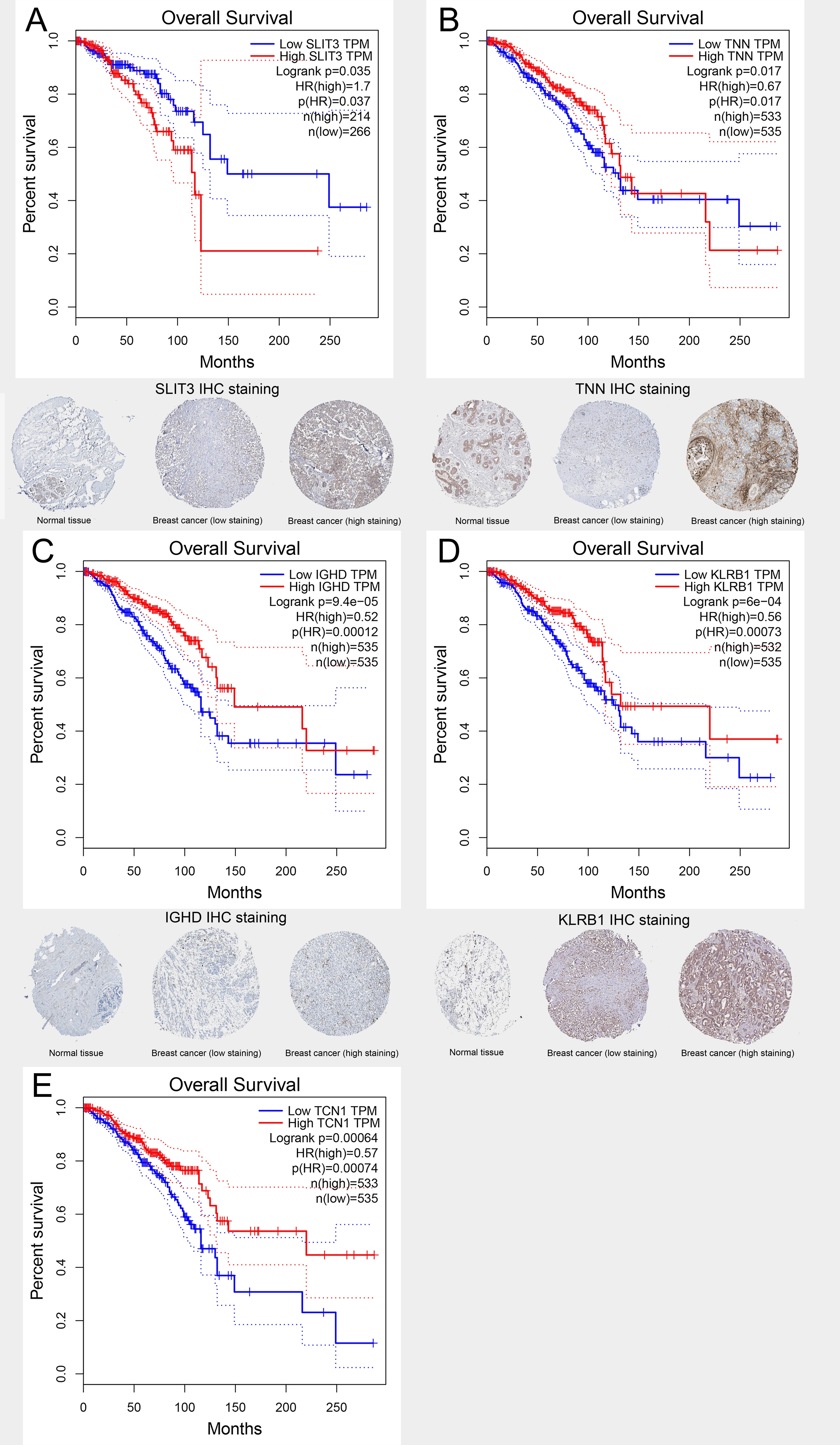

Supplement: Supplementary Figure 4 — Kaplan-Meier analysis and Immunohistochemical staining of the TME-risk signature, including SLIT3 (A), TNN (B), IGHD (C), KLRB1 (D), and TCN1 (E). TME; tumor microenvironment. [file Image_4.tif]

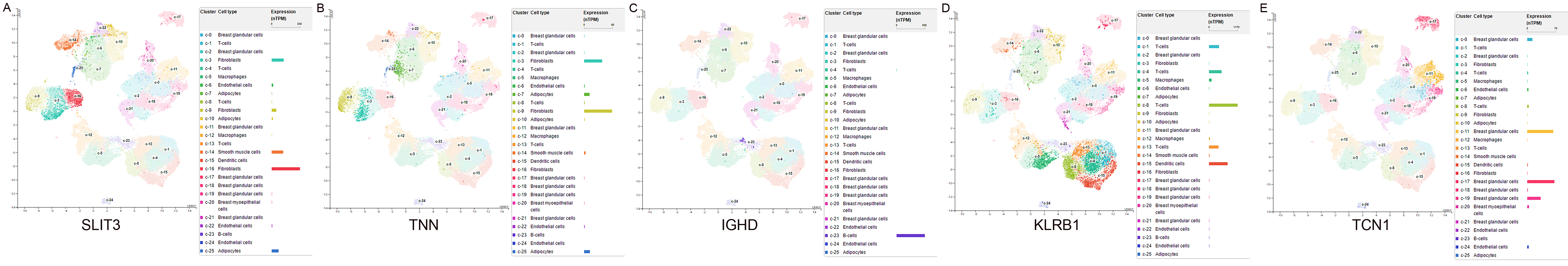

Supplement: Supplementary Figure 5 — The UMAP results of single cell sequencing of the TME-risk signature, including SLIT3 (A), TNN (B), IGHD (C), KLRB1 (D), and TCN1 (E). TME, tumor microenvironment. [file Image_5.tif]

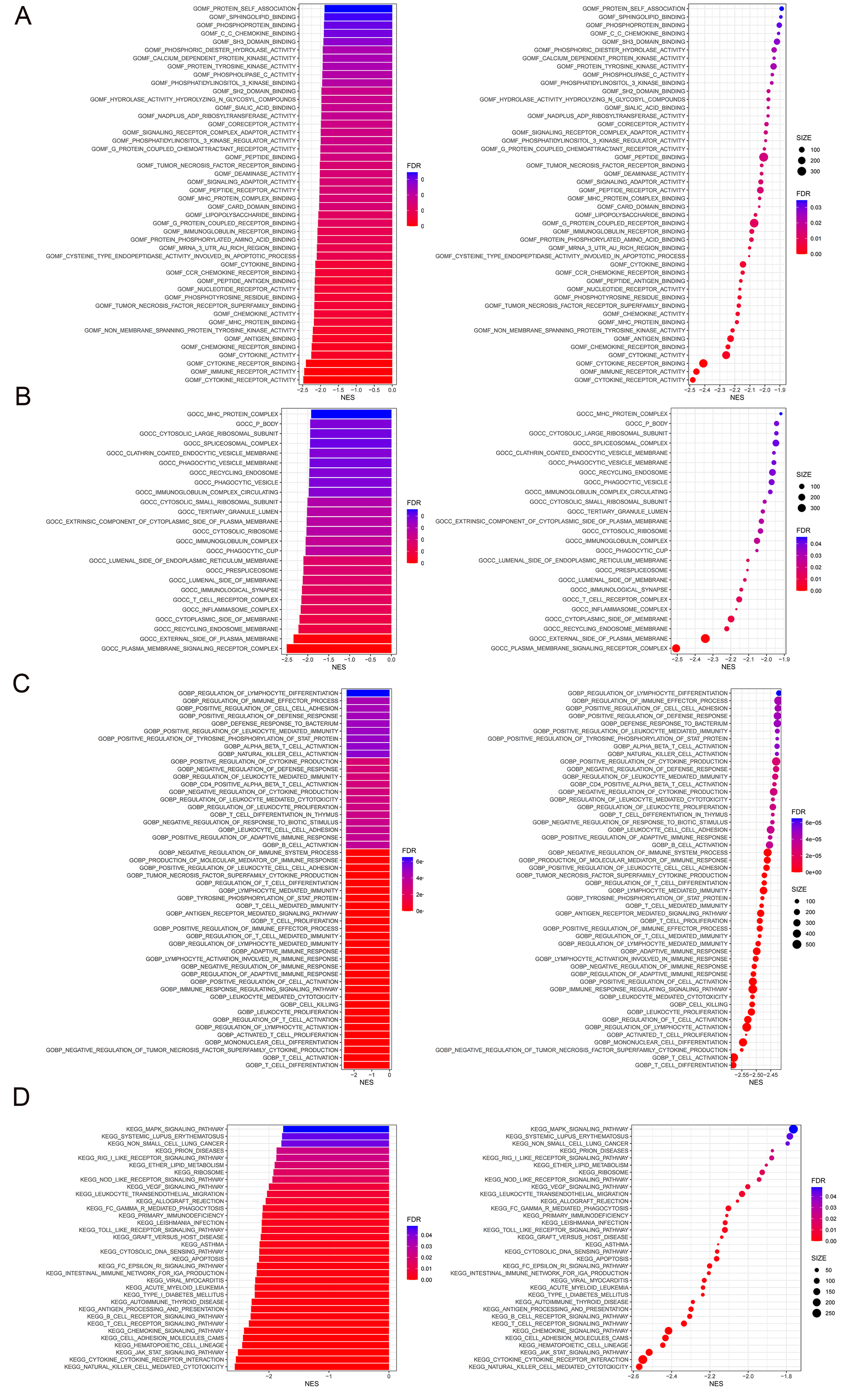

Supplement: Supplementary Figure 6 — GSEA analysis (including GO and KEGG analysis) between low and high TME-risk groups (top 50 listed biological function in BP (A), CC (B), MF (C) and KEGG (D), (FDR q-value <0.05 and |NES| > 1)). GSEA, gene set enrichment analysis; GO, gene ontology; KEGG, Kyoto Encyclopedia of Genes and Genomes. [file Image_6.tif]

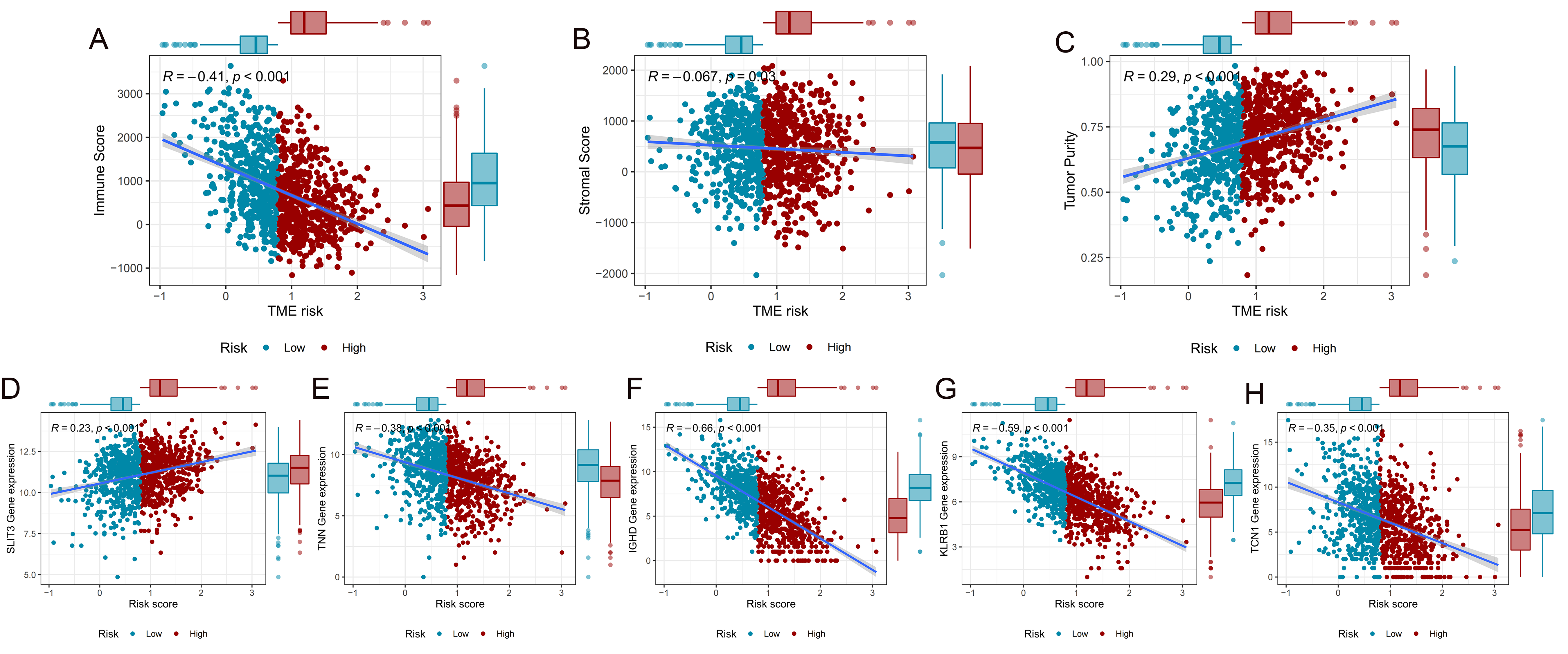

Supplement: Supplementary Figure 7 — Association between immune scores (A), stromal scores (B), tumor purity (C), and TME-risk scores and their distribution in the low and high TME-risk groups. Association between 5 screening genes (SLIT3 (D), TNN (E), IGHD (F), KLRB1 (G), TCN1 (H)) and TME-risk scores and their distribution in the low and high TME-risk groups. TME, tumor microenvironment. [file Image_7.tif]
